# University Marching Band Members’ Noise Dosages and Hearing Health-Related Knowledge

**DOI:** 10.3390/ijerph182111497

**Published:** 2021-11-01

**Authors:** Nilesh J. Washnik, Jeffrey A. Russell, Ishan Bhatt, Rebecca Meier, Olivia Chuzie, Nicole Nadeau, Shade Kirjava, Allyson Goff

**Affiliations:** 1 Hearing & Noise Research Lab, Department of Communication Sciences & Disorders, School of Rehabilitation & Communication Sciences, College of Health Sciences & Professions, Ohio University, W218 Grover Center, 1 Ohio University Drive, Athens, OH 45701, USA; 2 Division of Athletic Training, School of Applied Health Sciences & Wellness, College of Health Sciences & Professions, Ohio University, E182 Grover Center, 1 Ohio University Drive, Athens, OH 45701, USA; russelj4@ohio.edu; 3 Department of Communication Sciences & Disorders, University of Iowa, Iowa City, IA 52242, USA; ishan-bhatt@uiowa.edu; 4 Department of Communication Sciences & Disorders, School of Rehabilitation & Communication Sciences, College of Health Sciences & Professions, Ohio University, W218 Grover Center, 1 Ohio University Drive, Athens, OH 45701, USA; meierr@ohio.edu (R.M.); nn568618@ohio.edu (N.N.); ng402019@ohio.edu (A.G.); 5 Asheville Ear Nose and Throat, 1065 Hendersonville Rd., Asheville, NC 28803, USA; ochuzie@gmail.com; 6 Department of Population Health and Disease Prevention, University of California, Irvine, CA 92617, USA; skirjava@uci.edu

**Keywords:** noise dose, marching band, hearing health, performance, rehearsal, sound exposure, hearing protection

## Abstract

Objectives: (1) To measure sound exposures of marching band and non-marching band students during a football game, (2) to compare these to sound level dose limits set by NIOSH, and (3) to assess the perceptions of marching band students about their hearing health risk from loud sound exposure and their use of hearing protection devices (HPDs). Methods: Personal noise dosimetry was completed on six marching band members and the band director during rehearsals and performances. Dosimetry measurements for two audience members were collected during the performances. Noise dose values were calculated using NIOSH criteria. One hundred twenty-three marching band members responded to a questionnaire analyzing perceptions of loud music exposure, the associated hearing health risks, and preventive behavior. Results: Noise dose values exceeded the NIOSH recommended limits among all six marching band members during rehearsals and performances. Higher sound levels were recorded during performances compared to rehearsals. The audience members were not exposed to hazardous levels. Most marching band members reported low concern for health effects from high sound exposure and minimal use of HPDs. Conclusion: High sound exposure and low concern regarding hearing health among marching band members reflect the need for comprehensive hearing conservation programs for this population.

## 1. Introduction

High sound exposure among collegiate student musicians can exceed the recommended exposure limits specified by the National Institute for Occupational Safety and Health [1] on a daily basis [2,3,4,5,6,7,8]. Most of the hazardous sound exposure among these musicians occurs during rehearsals, individual practice, and other music activities [2,4,5,7]. Frequent exposure to high sound levels is associated with hearing loss [9,10,11,12] and with other hearing-loss-related symptoms, such as tinnitus, hyperacusis, and diplacusis [10,11,12,13,14]. Recent evidence suggests that about 40% of musicians report hearing difficulties [15]. Musicians have an approximately fourfold higher hazard ratio (HR) for NIHL and 57% higher HR for tinnitus when compared to the general population [12]. O’Brien et al. (2014) conducted a study on 600 musicians from eight Australian orchestras and found that 43% reported hearing loss [16]. Such a high prevalence of hearing loss in these musicians is a matter of serious concern because hearing is arguably a musician’s most valuable asset. Any loss of or damage to this sense modality has the potential to negatively affect a musician’s entire career [17].

Although several studies reported excessive noise exposure among student musicians playing different types of music [6,7,18], little is known with respect to sound exposure among marching band students and their hearing health risk perception due to loud sound exposure and preventive behaviors. The results of a study conducted by Russell and Yamaguchi (2018) revealed that the sound exposure levels and noise dose percentages among athletic trainers of marching bands greatly exceed the standards recommended by the National Institute of Occupational Safety and Health (NIOSH) [19]. These healthcare workers experienced high sound pressures while located on the periphery of their band’s playing, rather than when embedded within a band as a marching musician would be.

Considering the popularity of marching band and drum corps—particularly in high schools and universities—and the dearth of research on sound exposure and hearing health-related knowledge among marching band members, it is important to examine the sound exposure levels experienced by this type of musician during the performances, as well as their perception of hearing health risk. The purposes of this study were (1) to measure sound exposure of marching band and non-marching band students during a football game, (2) to compare these to sound level dose limits set by NIOSH, and (3) to assess the perceptions of marching band students about their hearing health risk from loud sound exposure and their use of hearing protection.

## 2. Materials and Methods

### 2.1. Participants

The university marching band maintained approximately 240 members during the season. The band’s instruments comprised of trumpets, trombones, mellophones, euphoniums, percussion (snares, timbales, tenors, basses, and cymbals), clarinets, alto saxophones, and tenor saxophones. The director of the marching band, 123 marching band students, and 2 non-marching band collegiate students who were placed in the football stadium’s audience volunteered for the study. The study protocol was approved by the university’s Institutional Review Board (IRB #18-X-337), and all participants provided their informed consent.

### 2.2. Sound Level Measurement during Rehearsals and Performances

The director of the marching band and six band members volunteered for sound level measurements. In addition, two students volunteered as audience members in the stadium during each football game. Among the six marching band members, one each played the trumpet, mellophone, percussion, trombone, alto saxophone, and clarinet. Sound exposure levels were measured with Etymotic ER-200D personal noise dosimeters (Etymotic Research, Elk Grove Village, IL, USA). The ER-200D noise dosimeter model has been previously applied in noise research [19,20,21] and found to be reliable as a measurement instrument [20]. The noise dosimeter measures sound levels using the A-weighting network. All the dosimeters were set according to the NIOSH standards with a threshold level of 75 dB, a criterion level of 85 dB, and an exchange rate of 3 dB [1].

The ER-200D collects noise data over 220 msec increments, sums the increments in 3.75 min blocks across the exposure duration, and stores these data in memory for a session record of 16 blocks per hour. The dosimeter produces equivalent continuous noise levels in dBA (L_Aeq_) and cumulative dose percentage values during the exposure period. In addition, we calculated the eight hour time-weighted average (TWA) sound levels (L_TWA(8)_). TWA is the overall noise exposure, determined by considering different exposure levels over various durations during the sound exposure period. L_TWA(8)_ was calculated using the following equation:

L_TWA(8)_ = (Q/Log_10_2) × [Log_10_(D/100)] + LC (1)

where Q = exchange rate (3 dB for NIOSH’s standards), D = noise dose, and LC = criterion exposure limit (85 dBA for NIOSH’s standards).

All the dosimeters were mounted at the chest level of six marching band members, the band director, and the two student audience members. Sound levels were collected from the musicians and the band director during two 90 min rehearsal sessions and two three-hour football game performances. Data from the participants stationed in the audience were collected during the football games for three hours each. The noise dosimeters were removed at the end of each of the rehearsal sessions and the football games. After each game, the students who volunteered to participate as audience members during the games informed the research team about their seat locations.

The noise dosimeters were connected to a computer, and measurements were downloaded. From these, we calculated 1 min averages, daily averages, and daily sound dose percentages according to the NIOSH criterion. Calculations were exported to Microsoft Excel for analysis.

### 2.3. Marching Band Members’ Risk Perception

Marching band members’ perceptions of noise exposure, the effect of sound levels on their hearing health and performance, and their protective behaviors were analyzed using a questionnaire (Supplemental Information S1). The questionnaire and an informed consent document were distributed to the 240 marching band members after the end of one of their rehearsal sessions. Of these, 123 marching band members (33 music majors and 90 non-music majors) returned their questionnaires with complete data. All the marching band members were informed that their participation was completely voluntary and that the results of the questionnaires would be used only for research purposes.

Rodrigues et al. [17] originally developed this questionnaire in Portuguese. A Portuguese language instructor from our university translated the questionnaire into English. Two faculty members, one from the Department of Athletic Training and one from the Department of Communication Sciences, provided content validation of the questionnaire. Some improvements related to English syntax were suggested and implemented in the English version of the questionnaire.

The questionnaire was divided into five parts. The first part of the questionnaire referred to students’ demographics, such as sex, age, major field of study, and year in school. In the second part of the questionnaire, questions pertaining to weekly exposure were included. These questions asked about the instrument(s) played by the student; hours of practice in different types of settings or classes; hours of individual and group rehearsals and exposure to other loud sound sources, which may include the use of headphones, attending concerts, and playing in other musical ensembles. The third part of the questionnaire analyzed the marching band members’ perceptions of sound levels with respect to (1) different types of classes, (2) group and individual practice sessions/rehearsals, and (3) different instruments (5-point Likert scale: 1 = very low; 5 = very high). The perceived effect of the sound levels on the participants’ performance also was analyzed (5-point Likert scale: 1 = not affected; 5 = greatly affected). 

The fourth part of the questionnaire consisted of seven questions designed to collect students’ views on the health effects of sound exposure. These questions asked students about their (1) general negative auditory and non-auditory effects of sound exposure on health (5-point Likert scale: 1 = not affected; 5 = greatly affected), (2) degree of concern about health effects (a list of health effects was presented and assessed on a 5-point Likert scale: 1 = no concern; 5 = very high concern), (3) previous hearing exams, and (4) ear symptoms (hyperacusis, diplacusis, tinnitus, sound distortion). In the last part of the questionnaire, marching band members were asked about measures for reducing sound levels, particularly regarding the use of hearing protective devices (HPDs) in different circumstances (5-point Likert scale: 1 = never use; 5 = always use). If students indicated their use of HPDs, they were asked about the type of hearing protection used. If HPDs were not used, the students were asked the reason for not using them. Participants also were asked about the use of instrument mutes (5-point Likert scale: 1 = never use; 5 = always use). An open-ended comment section was included at the end of the questionnaire.

Means and standard deviations were calculated for L_TWA(8)_ and noise dose for marching band rehearsal and performance. Sound exposure data from the rehearsals and performances were analyzed via analysis of variance (ANOVA). Levene’s test was utilized to assess the violation of homogeneity of group variances. For those noise exposure data with unequal group variances, differences in means between rehearsal and performance were evaluated with Welch’s modification of ANOVA to control for the unequal variances. Otherwise, the conventional ANOVA test was applied to the other variables. These statistical analyses were conducted using IBM SPSS version 25 (IBM Corp., Armonk, NY, USA) as were analyses of the questionnaire data.

## 3. Results

### 3.1. Sound Level Exposure

**Figure 1:**
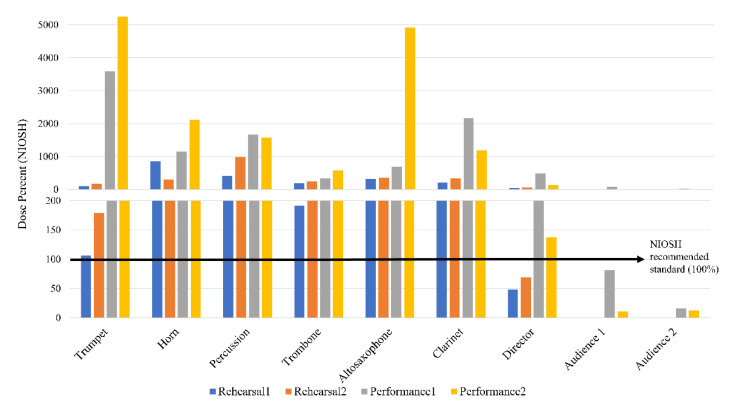
NIOSH noise doses during rehearsals and performances for participants as a function of instrument type.

**Figure 2:**
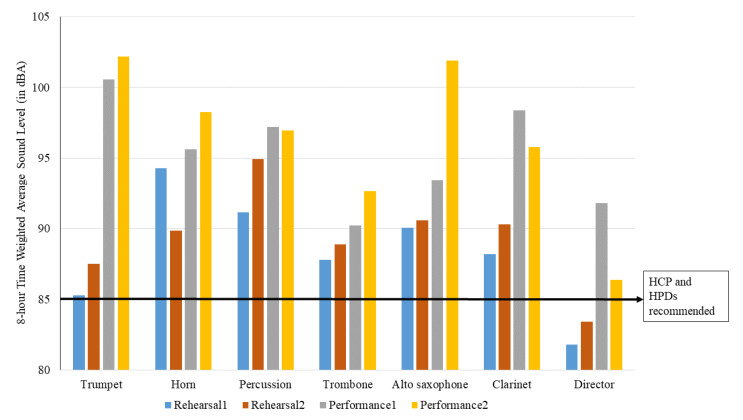
NIOSH 8 h time-weighted average sound levels for participants as a function of instrument type. Also shown are the levels at which NIOSH would recommend participation in a hearing conservation program (HCP) and the use of hearing protective devices (HPDs).

Our results demonstrated that all six marching band members exceeded 100% noise dose in each of the rehearsal sessions and game performances (Table 1 and Table 2, and Figure 1). The director of the marching band exceeded 100% noise dose only during game performances, while the noise dose of the audience members was below 100% during both of the football games. The data in Figure 1 and Table 1 and Table 2 also reveal that the noise dose was significantly higher during the performances than the rehearsals. Similarly, the L_TWA(8)_ was significantly higher among marching band members and directors during the performances compared to the rehearsals. Figure 2 shows NIOSH’s L_TWA(8)_ as a function of instrument type, as well as the levels at which the NIOSH would recommend a hearing conservation program (HCP) and the use of HPDs.

The result of Levene’s test revealed non-homogeneity of group variance for noise dose, but not for L_TWA(8)._ Thus, for the noise dose, Welch’s ANOVA indicated that the means among rehearsal and performance were significantly different for noise dose percentage (F_[1,13.71]_ = 11.563, *p* = 0.004). Standard ANOVA identified significant differences among the means of rehearsals and performances for L_TWA(8)_ (F_[1,26]_ = 19.80, *p* < 0.001). Pairwise differences between the means are provided in Table 3.

### 3.2. Marching Band Members’ Perception of Sound Exposure

Complete responses to the questionnaire were collected from the 123 marching band members. These participants were questioned about the effect of high sound level exposure on their performances. Thirty percent of them reported that high sound exposure does not affect their performance. On the other hand, 31.7% and 32.5% of the members believed that high sound exposure affected their performance slightly and moderately, respectively. Only 5.7% of the members identified a significant influence of loud music on their performance. Marching band members also were asked about the negative health effects associated with exposure to high sound levels. Only 10.6% of the members did not identify any negative health effects associated with high sound exposure, while 41.5% and 34.1% of members identified minimal and moderate negative effects of high sound exposure on health, respectively. Nearly 24% of the musicians did not show any concern for the auditory effects of sound exposure such as hearing loss, tinnitus, diplacusis, hyperacusis, and distortion.

**Figure 3:**
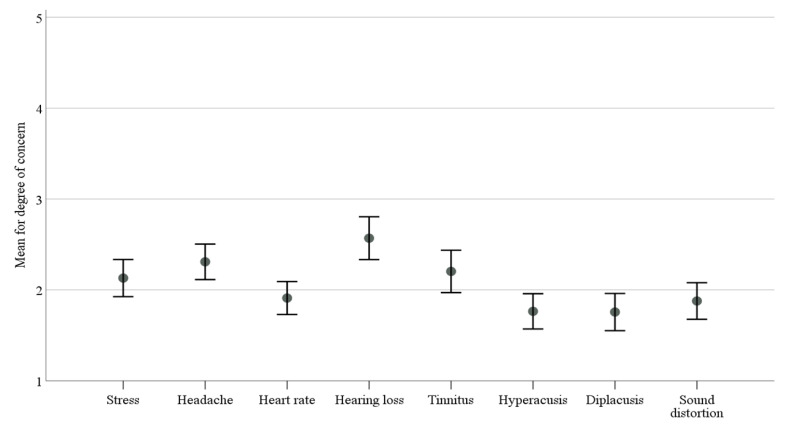
Degree of concern with health effects.

Figure 3 and Table 4 show the participants’ degree of concern with different health effects. Overall, marching band members indicated low concern in relation to health effects that may accompany high sound exposure. Higher levels of concern were identified for stress, headache, increased heart rate, hearing loss, and tinnitus. It is also evident from Table 4 that a significant number of students showed no concern for various related health effects, particularly for tinnitus (42.3%), hyperacusis (57.7%), diplacusis (60.2%), and sound distortion (52%). Moreover, only 8.1% of the participants reported frequent use of hearing protection. The majority of the marching band members either reported occasional use of hearing protection or no use of hearing protection at all. 

## 4. Discussion

### 4.1. Sound Level Exposure

The purposes of this study were (1) to measure sound exposure of marching band and non-marching band students during a football game, (2) to compare these to sound level dose limits set by NIOSH, and (3) to assess the perceptions of marching band students about their hearing health risk from loud sound exposure and their use of hearing protection. Our results suggest that during both rehearsals and performances, marching band members are exposed to sound levels that could put them at risk for developing noise-induced hearing loss (NIHL). During a typical fall semester, the members of the band perform during six football games, which include five home football games and one professional football game. The marching band members also are required to rehearse five times a week for 13–15 weeks, and each rehearsal session lasts for 1.5 h. The rehearsal sessions comprise both music-only practice and marching while playing.

During rehearsals, all marching band members, excluding the director, were subjected to sound levels greater than NIOSH’s 85 dBA standard. The noise dose data of this study show that the mean noise dose during rehearsals was 330.38% among marching band members. Such high noise exposure on a regular basis may have a hazardous effect on the hearing of marching musicians.

During both performances, all six marching band members and the director exceeded NIOSH’s 100% noise dose. The mean noise dose and L_TWA(8)_ during the performance were 1847.42% and 95.81 dBA, being significantly higher during performances than rehearsals. This finding is consistent with previous reports [19,22]. The higher sound levels during performances might be related to factors such as performance related arousal, music performance anxiety, feedback from the audience, changes in the acoustic environment, and the dynamic demands of the marching band director. Considering the frequency of both performances and rehearsals for marching band members and the hazardous noise dose that these members experience, this places marching band members at very high risk for NIHL.

The noise dose of the director of the marching band was below 100%, and his L_TWA(8)_ was below 85 dBA in both rehearsal sessions. In contrast, all six marching band members exceeded the 100% noise dose and 85 dBA L_TWA(8)_ limit during the two rehearsal sessions. This difference is attributed to the distance between the marching band director and the marching band. To see the marching band formation and to conduct the instrumentalists, the marching band director stood approximately 25 m away and was elevated on a platform during the rehearsals. The distance between the marching band director and the marching band was less than 5 m during performances; this resulted in a higher noise dose (exceeding 100%) and higher L_TWA(8)_ for the director during performances. Thus, it can be implied that marching bands, including their directors, are exposed to more hazardous sound levels during performances than during rehearsals.

The noise dose and L_TWA(8)_ among the audience members during the two performances were well below 100% and 85 dBA. This finding is not consistent with the previous studies [23,24,25]. The low noise exposure among audience members could be attributed to two factors. First, the distance between the marching band and the audience members during the performances was approximately 30 to 40 m. According to the inverse square law, the intensity of sound drops significantly after covering that distance. Second, the size of the audience during the two football games may have played a role in the sound levels. The number of people attending the games was 13,774 and 12,938. This is a relatively small crowd compared to football games at large universities and the National Football League. Crowd size is one of the important factors that can influence noise levels during sports contests [26]. To our knowledge, this is the first study to compare sound exposure levels among marching band members, the director of the marching band, and the audience at an American football game and to analyze the auditory risk perception among marching band members. In light of our results, and given the high sound levels we measured, all marching band members are at risk for developing NIHL during both rehearsals and performances.

Russell and Yamaguchi [19] measured sound pressure levels among eight athletic trainers working with a marching band at outdoor rehearsals, indoor field house rehearsals, and outdoor performances. They collected sound data using the same dosimeters (Etymotic ER-200D) as used in this study, which were also set at the NIOSH standards of 85 dBA and 3 dB exchange rate. The event duration of various rehearsal and performance sessions ranged from 11 min to 6 h 50 min. The results of their study suggest the same conclusion as ours: (1) marching band sound exposure exceeds safe levels during rehearsal and performances and (2) noise dose is significantly higher in performances than in rehearsals. The noise dose for marching band athletic trainers reported by Russell and Yamaguchi [19] during rehearsals and performances ranged from 2% to 93% and 2% to 557%, respectively. Their low minimum values were likely due to some of their noise measurements not being conducted during football games; they also recorded sound levels at parades and other non-athletic band performances. The noise dose in the present study among marching band members (including the director) for rehearsals and performances ranged from 48 to 981% and 137 to 5252%, respectively. The reason our study recorded higher noise doses is that we collected our measurements from marching band musicians who were playing inside a typical band block formation. Thus, musicians being in close proximity to other musicians concentrates sound levels.

The sound exposure of marching band members could have been influenced by other factors, such as the position of marching band members and the hearing status of the marching band director. Previous studies have shown that the position of members in a band has a significant influence on the amount of noise exposure/noise dose [27]. Henoch and Chesky [28] studied the effect of positions on sound exposure levels among members of jazz band ensemble and reported that musicians who are surrounded by other musicians are exposed to comparatively higher sound levels than musicians on the perimeter of the band. Marching band members in close proximity to other loud musical instruments such as horn or drum may expose to higher sound levels than their peers who are not in close proximity to these loud musical instruments [7]. The position of marching band members changes constantly during rehearsals and performances because of different marching band formations and transformations. Thus, it is very difficult to control the effect of position on the amount of sound exposure among marching band members.

Moreover, the hearing acuity of the director may have a significant influence on the sound levels produced by marching band members. For example, a marching band director with significant hearing loss may demand a higher level of compressed dynamics that may result in higher sound levels during marching band rehearsals and performances. Unfortunately, the hearing sensitivity of the marching band director and members were not measured in this study. Thus, it is difficult to rule out the influence of the director’s hearing on the sound levels generated by the marching band members.

### 4.2. Marching Band Members’ Perceptions of Hearing Health Risk

Unfortunately, despite these alarming sound exposure results, our study suggests that the participating marching band members do not appear concerned about their exposure to high sound levels. A considerable number of marching band members were not bothered about the influence of high sound exposure levels on their performance. Their level of worry about other negative hearing health effects, such as hearing loss, hyperacusis, and stress, also was low. Approximately one-fourth of the marching band members showed no concern for any of the hearing health risks associated with high sound exposure.

Our data also indicate that 92% of marching band members are reluctant to use hearing protection. Less than 10% of the musicians reported using such protection frequently. A literature review suggests that musician reports very limited use of HPDs due to pain; pressure; discomfort; HPDs’ inference with playing and monitoring ability; and distortion of timbre, dynamics, and sonority when they and their peers are playing [13,29,30,31,32,33]. To cater to the hearing protection needs of the musicians, HPD manufacturers developed musicians’ ear plugs (MEPs), claiming that MEPs replicate the natural response of the ear canal and offer adequate attenuation without compromising the spectrum of music and listening quality [33]. However, Chesky and Amlani [32] found that the claims used to market MEPs to musicians are misleading. They reported discrepancies for claiming attenuation characteristics in response to musical sounds and attributed these, in part, to the manufacturers’ testing procedures for measuring attenuation and listening quality [32].

Indifference to the impact of high sound exposure and associated health effects on marching musicians might also be responsible for these individuals’ limited use of hearing protection. Bohlin and Erlandsson [33] noted that if young adults identify themselves as susceptible to negative consequences of loud sound exposure, they are more willing to adopt preventive behavior. Otherwise, risky behaviors are adopted. Our outcomes differed from the results of studies with professional musicians [14,34]. More importantly, Miller et al. [2] reported that 22% of student musicians wear hearing protection while playing their musical instruments. The results of the present study suggest that marching band students need improved understanding that high sound exposure may result in hearing loss, tinnitus, diplacusis, hyperacusis, and distortion, even at a young age. Marching band members also should be apprised that, although hearing aids can help in instances of hearing loss, they do not restore hearing loss. Decidedly, then, the best strategy for musicians is prevention of hearing loss.

Our results suggest the need to provide more information about hearing conservation and to promote healthy hearing behavior among marching band members. University-based hearing conservation programs for student musicians and marching band members not only educate students about NIHL but also increase the use of HPDs among them [35]. Possible strategies in promoting healthy hearing behavior could include simulated hearing loss for extended periods, improved education on noise exposure and its auditory and non-auditory effects on health, and improved availability of HPDs with real flat attenuation across the frequency range [36]. All of these strategies can be implemented efficiently through a university-level hearing conservation program for student musicians and marching band members.

Limitations of the present study include the inability to index changes in the hearing thresholds due to exposure to loud music generated by the marching band. We did not measure pre-and post-exposure hearing thresholds of the marching band members because this was not our research objective in this study. Another limitation of the present study is that the results of this study are based on the measurement and survey of one marching band. This marching band may not be representative of marching band members as a population. Future studies can investigate changes in hearing thresholds among marching band members after rehearsal and performance sessions.

## 5. Conclusions

The results of this study suggest that marching band members are exposed to hazardous sound levels during marching band rehearsals and performances. Such high sound exposure among marching band members puts them at risk for NIHL. Despite such hazardous sound exposure, it was found that marching band members are minimally concerned about the effects of high sound exposure on their performance and health. Our results support the need for comprehensive hearing conservation programs for marching band members that should include sound level monitoring; annual hearing evaluation; education on the impact of high sound exposure on hearing, health, and performance; and the use of hearing protection. If marching band musicians and directors do not receive hearing conservation services, they will continue to be at an increased risk for developing NIHL.

## Figures and Tables

**Table 1:** Summarized data for noise dose, L_Aeq_ range, and L_TWA(8)_ of 6 marching band members and director during rehearsals.

	Rehearsal 1			Rehearsal 2		
Instrument	Noise Dose (%)	L_TWA(8)_ (dBA)	L_Aeq_ Range (dBA)	Noise Dose (%)	L_TWA(8)_ (dBA)	L_Aeq_ Range (dBA)
Trumpet	106.6	85.27	70.3–98.2	179.0	87.52	61.0–100.0
Horn	848.9	94.28	76.5–109.5	307.2	89.87	70.9–100.6
Percussion	414	91.17	79.3–105.0	981.3	94.91	79.5–105.8
Trombone	191.5	87.82	67.0–101.1	245.4	88.89	72.4–100.7
Alto saxophone	321.4	90.07	76.2–102.7	361.6	90.58	71.7–101.8
Clarinet	209.8	88.21	70.0–103.7	341.2	90.33	73.0–103.2
Director	48	81.81	76.6–97.7	69.5	83.41	78.3–94.6

L_TWA(8)_—the eight hour time-weighted average (TWA) sound levels; L_Aeq_—the equivalent continuous noise levels in dBA.

**Table 2:** Summarized data for noise dose, L_Aeq_ range, and L_TWA(8)_ of 6 marching band members, director and non-marching band students during performances.

	Performance 1			Performance 2		
Instrument	Noise Dose (%)	L_TWA(8)_ (dBA)	L_Aeq_ Range (dBA)	Noise Dose (%)	L_TWA(8)_ (dBA)	L_Aeq_ Range (dBA)
Trumpet	3588.4	100.54	94.0–111.0	5252.1	102.20	91.2–112.3
Horn	1153.4	95.61	83.1–106.0	2120.0	98.26	77.7–107.6
Percussion	1666.7	97.21	78.5–106.4	1575.6	96.97	82.0–108.6
Trombone	333	90.22	70.7–101.8	582.9	92.65	78.1–101.4
Alto saxophone	695.9	93.42	77.7–104.5	4919.0	101.91	73.5–114.1
Clarinet	2165.9	98.35	80.7–112.7	1193.4	95.76	70.0–108.7
Director	480.3	91.81	0.0–101.7	137.4	86.37	74.5–100.3
Audience 1	81.9	84.13	75.5–99.4	10.6	75.25	65.1–84.6
Audience 2	16.2	77.09	70.4–89.4	12.6	76.00	72.2–88.0

L_TWA(8)_—the eight-hour time-weighted average (TWA) sound levels; L_Aeq_—the equivalent continuous noise levels in dBA.

**Table 3:** Minima, maxima, means, and standard deviations of sound exposure among marching band members.

		Rehearsals	Performances	Significant Difference
Number of observations		14	14	
L_TWA(8)_	Min	81.81	86.37	
	Max	94.91	102.20	
	Mean ± SD	88.87 ± 3.66	95.81 ± 4.54	**
Dose %	Min	48	137.4	
	Max	981.3	5252.10	
	Mean ± SD	330.38 ± 272.18	1847.42 ± 1646.92	**

** significantly different at *p* < 0.005.

**Table 4:** Percent of respondents expressing the level of concern with health effects among marching band members.

	None	Low	To a Certain Degree	High	Very High
Stress	39.0	25.2	23.6	8.1	4.1
Headache	28.5	29.3	28.5	10.6	3.3
Heart rate	43.9	30.9	17.9	4.9	2.4
Hearing loss	29.3	20.3	23.6	17.9	8.9
Tinnitus	42.3	21.1	18.7	9.8	8.1
Hyperacusis	57.7	20.3	13.0	5.7	3.3
Diplacusis	60.2	19.5	9.8	5.7	4.9
Sound distortion	52.0	22.0	16.3	5.7	4.1

## Data Availability

The datasets used and/or analysed during the current study are available from the corresponding author on reasonable request.

## Supplementary Materials

The following are available online at https://www.mdpi.com/article/10.3390/ijerph182111497/s1, Supplementary material S1: PDF title ‘Analysis of the Sound Exposure and Risk Perception in Musical Practices Questionnaire’ submitted as supplemental document.
