# Supplementary material for: University Marching Band Members’ Noise Dosages and Hearing Health-Related Knowledge
Source: Int J Environ Res Public Health. 2021 Nov 1;18(21):11497. doi: 10.3390/ijerph182111497 (PMC8583652; doi:10.3390/ijerph182111497)
Supplement: Supplementary file 1 [file ijerph-18-11497-s001.zip › ijerph-1415223-supplementary.pdf]

Supplementary Material: Questionnare

Analysis of the Sound Exposure & Risk Perception in Musical Practices

Your participation is essential to this study. Read all the questions carefully and answer as requested. The retrieved data are absolutely confidential, and the participant's identity will not be disclosed.

General Information: **ID No.____________**

1. **Age:** ___ years old
2. **Sex**: ◯ Male ◯ Female ◯ Prefer not to answer
3. **Department/Program (major)** : ______________________
4. **Year in school** : ◯ Fr ◯ So ◯ Jr ◯ Sr ◯ 5th ◯ Grad

Instrument and hours of practice

5. **How long have you been actively involved in the marching band?** _________ year/s_____ months
6. **What is the primary instrument that you play?** _________________________
7. **Do you play any other instruments?** Yes ◯ No◯
   a. If yes, which one(s): _______________________
8. **How many average hours do you spend, per week, practicing the following:**
   a. Private lessons ____ hours
   b. Chamber music lessons ____ hours
   c. Combo classes ____ hours
   d. Orchestra lessons ____ hours
   e. Choir lessons ____ hours
   f. Other group classes (e.g. auditory training) ____ hours
   g. Individual practice outside of classrooms (e.g. home) ____ hours
   h. Music study group outside of classrooms ____ hours
9. **Are you involved with other activities, music related or not, where you are exposed to loud noises outside of classes?**

   Yes ◯ No ◯

   9.1. If yes, check the appropriate boxes and write how many hours are spent weekly with each activity:
   - ◯ Participate in marching bands, orchestras or others ____ hours
   - ◯ Watch concerts ____ hours
   - ◯ Listen to music from headphones ____ hours
   - ◯ Go to night clubs ____ hours
   - ◯ Other activities: _______________ ____ hours

Musical practices and sound level exposure

10. **How do you rate the sound level in the following practices?**

	Very low	Low	Average	Loud	Very loud
Private lessons	◯	◯	◯	◯	◯
Chamber music lessons	◯	◯	◯	◯	◯
Combo classes	◯	◯	◯	◯	◯
Orchestra lessons	◯	◯	◯	◯	◯
Choir lessons	◯	◯	◯	◯	◯
Other group classes	◯	◯	◯	◯	◯
Individual practice	◯	◯	◯	◯	◯
	◯	◯	◯	◯	◯

Appendix- I

Analysis of the Sound Exposure & Risk Perception in Musical Practices

Marching band practices

11. How do you rate the sound levels emitted by the following instruments?

	Very low	Low	Average	Loud	Very loud
String instruments	◯	◯	◯	◯	◯
Woodwind instruments	◯	◯	◯	◯	◯
Brass instruments	◯	◯	◯	◯	◯
Percussion and timpani	◯	◯	◯	◯	◯
Pianos/Organ	◯	◯	◯	◯	◯
Other: ____________	◯	◯	◯	◯	◯

12. Do you believe that being exposed to loud noises affects your performance in music?

No ◯ A Small amount ◯ A Moderate amount ◯ A Lot ◯

Health

13. Do you believe that being exposed to loud noises is harmful to your health?

No ◯ A Small Amount ◯ A Moderate Amount ◯ A Lot ◯

14. How do you rate your level of concern about the occurrence of the following health threats as a result of exposure to high levels of noise during musical practices?

	None	Low	Moderate	High	Very high
Stress	◯	◯	◯	◯	◯
Headaches	◯	◯	◯	◯	◯
Increase in heart rate	◯	◯	◯	◯	◯
Hearing loss	◯	◯	◯	◯	◯
Tinnitus	◯	◯	◯	◯	◯
Hyperacusis*	◯	◯	◯	◯	◯
Diplacusis**	◯	◯	◯	◯	◯
Distortion	◯	◯	◯	◯	◯

*Hyperacusis is described as a heightened sensitivity to sound, such that even “low” levels of sound may cause pain and discomfort

** Diplacusis: An aural deficiency in which the PITCH of a single TONE is heard doubly (i.e., as two different pitches) by the two ears.

15. Have you ever completed any hearing exams? Yes ◯ No ◯

If yes, when was the last one?

This year ◯ 1-3 years ago ◯ 4-5 years ago ◯ 6-10 years ago ◯

More than 10 years ago ◯ I do not remember ◯

Appendix- I

Analysis of the Sound Exposure & Risk Perception in Musical Practices

16. Have you ever had tinnitus? (Constant ringing in ears or head even when there are no noises around.)

Yes ◯ No ◯

Note: *If you ever had tinnitus, please answer the next couple of questions. If not, skip to question 17.*

16.1. Is it always present? Yes ◯ No ◯

16.2. Have you ever had tinnitus after one of the following practices?

	Never	Rarely	Sometimes	Often	Always
Private lessons	◯	◯	◯	◯	◯
Chamber music lessons	◯	◯	◯	◯	◯
Combo classes	◯	◯	◯	◯	◯
Orchestra lessons	◯	◯	◯	◯	◯
Choir lessons	◯	◯	◯	◯	◯
Other group classes	◯	◯	◯	◯	◯
Individual practice	◯	◯	◯	◯	◯
Marching band practices	◯	◯	◯	◯	◯
Other activity:___________	◯	◯	◯	◯	◯

17. Have you ever had hyperacusis? (Abnormal sensitivity to everyday sounds and noises. Many times there is also sensitivity to sharp sounds.)

Yes ◯ No ◯

17.1. If yes, which ear was affected?

Right ◯

Left ◯

Both ◯

18. **Have you ever experienced any sound distortion?** (When the sound reaches a level that feels abnormal or distorted.)

Yes ◯ No ◯

19. **Have you ever had diplacusis?** [Diplacusis: An aural deficiency in which the PITCH of a single TONE is heard doubly (i.e., as two different pitches) by the two ears.]

Yes ◯ No ◯

Reduction of sound level and hearing protection

20. Do you ever wear any hearing protection during the following practices?

	Never	Rarely	Sometimes	Often	Always
Private lessons	◯	◯	◯	◯	◯
Combo classes	◯	◯	◯	◯	◯
Orchestra lessons	◯	◯	◯	◯	◯
Individual practice	◯	◯	◯	◯	◯
Music study group	◯	◯	◯	◯	◯
	◯	◯	◯	◯	◯

Appendix- I

Analysis of the Sound Exposure & Risk Perception in Musical Practices

Participating in marching bands

Other: ________________

20.1. If you do wear hearing protection, which one(s) do you usually use?

- Earplugs ○
- Acoustic earmuffs ○
- Musicians earplugs ○
- Cotton ○
- Other: ______________ ○

20.2. If you do not wear hearing protection, what is the reason? (more than one box can be checked)

- I have never tried them on ○
- I do not think they are necessary ○
- They are uncomfortable ○
- I do not know which protection to use ○
- They affect my performance ○
- My instrument does not sound the same ○
- They make it difficult to hear my instrument's sound ○
- They are difficult to put on ○
- They cause ear infections ○
- They make me dizzy ○
- Other: ______________ ○

21. **How often do you use mute on your instruments?**

Never ○ Rarely ○ Sometimes ○ Frequently ○ Always ○

22. **Do you usually do anything else to reduce sound level exposure when you play?**

Yes ○ No ○

22.1. If yes, what do you usually do? ____

Comments or suggestions:

____

____

____

____

Thank you for your participation!
